# Polyphony: superposition independent methods for ensemble-based drug discovery

**DOI:** 10.1186/1471-2105-15-324

**Published:** 2014-09-30

**Authors:** William R Pitt, Rinaldo W Montalvão, Tom L Blundell

**Affiliations:** Department of Biochemistry, University of Cambridge, 80 Tennis Court Road, Cambridge, CB2 1GA UK; UCB Pharma, 208 Bath Road, Slough, Berkshire, SL1 3WE UK; University of São Paulo,São Carlos Institute of Physics, Av. Trabalhador são-carlense, 400 - Pq. Arnold Schimidt, São Carlos, CEP: 13566-590 SP Brazil

## Abstract

**Background:**

Structure-based drug design is an iterative process, following cycles of structural biology, computer-aided design, synthetic chemistry and bioassay. In favorable circumstances, this process can lead to the structures of hundreds of protein-ligand crystal structures. In addition, molecular dynamics simulations are increasingly being used to further explore the conformational landscape of these complexes. Currently, methods capable of the analysis of ensembles of crystal structures and MD trajectories are limited and usually rely upon least squares superposition of coordinates.

**Results:**

Novel methodologies are described for the analysis of multiple structures of a protein. Statistical approaches that rely upon residue equivalence, but not superposition, are developed. Tasks that can be performed include the identification of hinge regions, allosteric conformational changes and transient binding sites. The approaches are tested on crystal structures of CDK2 and other CMGC protein kinases and a simulation of p38α. Known interaction - conformational change relationships are highlighted but also new ones are revealed. A transient but druggable allosteric pocket in CDK2 is predicted to occur under the CMGC insert. Furthermore, an evolutionarily-conserved conformational link from the location of this pocket, via the αEF-αF loop, to phosphorylation sites on the activation loop is discovered.

**Conclusions:**

New methodologies are described and validated for the superimposition independent conformational analysis of large collections of structures or simulation snapshots of the same protein. The methodologies are encoded in a Python package called Polyphony, which is released as open source to accompany this paper [http://wrpitt.bitbucket.org/polyphony/].

## Background

Researchers carrying out structure-based drug design (SBDD) are constantly looking to improve the modelling of protein conformational change and its relationship to ligand binding. It is well known that protein-target conformational flexibility can lead to problems in, for instance, small molecule binding-mode prediction and structure-activity relationship interpretation [1, 2]. It has been said that a lack of appreciation of the dynamics of macromolecular complexation is holding back progress in virtual screening [3]. Changes in protein conformation, when experimentally observed, can lead to the discovery of highly prized cryptic binding sites [4] and allosteric pockets [5, 6]. For the discovery of protein-protein interaction inhibitors, where there is no small endogenous small molecule to mimic, the treatment proteins as flexible entities is especially important [7].

NMR is perhaps the experimental technique most able to report on protein structure and dynamics in solution. However, multiple crystal structures of the same protein can provide much information on the conformational alternatives adopted by protein structures when interacting with other molecules [8]. X-ray crystal structures are the basis of the majority of SBDD projects, and drug companies often amass hundreds of crystal structures of the same protein with different ligands bound over the course of a drug discovery project. It has been estimated that the pharmaceutical industry as a whole currently solves 10,000 macromolecular crystal structures each year [9]. Normally only one set of model coordinates is provided per structure solution but many similar models can be found that fit the experimental data equally well [10]. Single sets of structure coordinates can also be used to generate ensembles using conformational sampling techniques [11] or simulations [12]. Taken together, experimental and computer generated ensembles should provide a fuller picture of a protein molecule’s true nature [2]. Many authors have encouraged the use of protein-structure ensembles in drug discovery [13–16]. Here we would like to distinguish this approach from more traditional (single) SBDD by the coining the expression “ensemble-based drug discovery” (EBDD).

It is still not straightforward with existing molecular modelling and bioinformatics tools to make full use of the large and ever increasing number of structures in some drug target families in the PDB [17] and in proprietary collections. Software for the analysis of structural ensembles is readily available of course. Root mean squared deviation (RMSD) of equivalent atoms, after optimal superposition, is the most widely used measure of pairwise protein structure similarity. Similarly root mean squared fluctuation (RMSF) is used to measure residue positional variability. Principal component analysis (PCA), or essential dynamics, is a very effective way of distilling the most important motions from molecular dynamics (MD) trajectories [18], and structures derived from X-ray crystallography [19] and NMR [20]. Many programs provide functionality for their calculation, for example GROMACS analysis modules [21], Bio3D [22] and Dynamite [23]. Wordom [24] is a package for the analysis of MD simulations which also provides PCA, along with many other analysis techniques. Many software packages designed for the analysis of MD snapshots require a consistent set of atoms and residues, making their use on crystal structures awkward. Comparisons of the essential dynamics produced by MD with principal components derived from crystal and NMR structures have been used to validate the results of the former approach [19, 20]. ProDy [25] facilitates the comparison of crystal structure and MD trajectory PC’s with normal modes calculated from a single structure. Other programs allow pairwise comparison of protein structures in order to identify hinge regions and interdomain motions. These include MolMovDB [26], DynDom [27], FlexProt [28] and FATCAT [29]. Dihedral angle PCA [19, 30] is a less common approach but can be used to identity hinge regions in proteins [19]. Another technique employed is distance difference matrices, for example using STRUSTER [31]. As well as the coordinates of a protein, one can also study the pockets formed by its surface and how they change as the protein conformation changes. These pockets are of particular interest to those involved in drug discovery. MDPocket is designed for the analysis of pockets in MD simulations using Fpocket [32]. ProVar [33] uses calculated surface properties, generated by a range of programs, assigned to individual residues allowing comparison between the results and within ensembles of structures without superposition.

The techniques developed here are designed to complement the existing approaches described above, especially those employing RMSD and coordinate PCA that rely upon molecular superposition. Molecular superposition becomes problematic when large conformational changes occur in a protein, involving multiple domains and RMSD can violate the triangle inequality rule [34]. In such cases it is useful to compare the curvature and torsion of a spline fitted to Cα atoms, an approach that was first used for the comparison of protein backbone conformations in 1978 [35]. Although the uses of this and related approaches (e.g. Chang *et al.* [36]) remain relatively uncommon, curvature and torsion of Cα splines have been utilised to find the conserved core of homologous proteins [37], to analyse secondary structure motifs [38] and to assess structural alignments of distantly related proteins [39]. This sort of analysis is very efficient and scales well with the size and number of structures in an ensemble.

In the work described here, Cα-spline curvature and torsion, together with side-chain conformation, intermolecular interaction fingerprints and pocket properties, are represented as per-residue descriptors and grouped by alignment position in a way that is analogous to correlated mutation analysis sequence analysis techniques [40]. Summary statistics and inter-residue relationships calculated in this way are mapped onto representative 3D structures to aid visual interpretation of the results. All the methodologies are programmed in a purpose built python package called Polyphony, in which the “plug-in” architecture allows other descriptors of protein structure generated by 3rd party programs, for example Fpocket [32], NCONT [41], Credo [42] and Piccolo [43], to be added with very little effort. Matplotlib [44], PyMol [45], Jalview [46], and ETE [47] are used for visualisation of the results.

Below various novel metrics and algorithms are described for the statistical analyses of local geometry of proteins, compared across ensembles of structures. These include a tailor-made variance measure that is used to distinguish random thermal-like fluctuations from significant conformational changes. In addition, existing techniques such as PCA are applied in new ways to differential geometry descriptors of protein conformation. A novel approach to using the output from Fpocket [32] to identify distinct and cryptic pockets from an ensemble of structures is described. Due to the scalability and automation of the general approach taken it can be applied to large sets of experimental structures or calculated conformations. These advantages are illustrated below by a comparison of conformational commonalities within evolutionary related proteins and by comparing snapshots from an MD simulation with X-ray crystal structures of the same protein.

The ultimate aim of this work is to extend the repertoire of tools available to those wanting to do ensemble-based drug discovery. However, it is hoped that the methods will also be used to discover fundamental mechanisms in the makeup of protein machines. All the known mechanistic changes in CDK2 and related structures were discovered afresh but in addition, more subtle, evolutionarily conserved allosteric changes were revealed.

## Results and discussion

**Figure 1:**
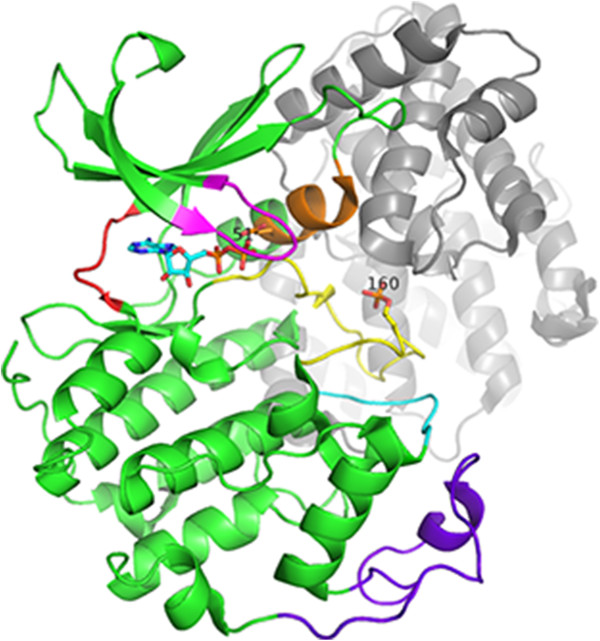
**The structure of the CDK2 - cyclin complex (3QHR [**[60]**]).** This structure is phosphorylated at Thr 160, and bound to cyclin A2 (grey) and ADP (sticks). The PSTAIRE region is shown in orange, the T-loop in yellow, the glycine rich loop in magenta, the CGMC insert in purple, the αEF-αF loop in cyan and the hinge region in red.

The initial emphasis of Polyphony is on the analysis of structures from X-ray crystallography but it can be used on NMR structures and snapshots along the trajectories of protein simulations. Cyclin-dependent kinase 2 (CDK2) is often used to validate computational approaches which treat protein-ligand complexes as flexible entities (e.g. [48–53]) due to the large number of crystal structures deposited in the PDB [17] and to the conformational changes that can be observed in these structures. It is a protein kinase involved in cell cycle progression and inhibitors of its function have been designed with the purpose of exploring their utility as anticancer agents [54, 55]. Kinase activity is switched on by binding of cyclin and phosphorylation by CDK activating kinase (CAK) [56]. CDK2 is inhibited by the binding of Cip and INK4 family proteins [57]. The binding of cyclin A or cyclin E results in conformational change in which the PSTAIRE region (see Figure 1) rotates and swings in so that the Glu 51 takes its place as part of the catalytic triad [57, 58]. The N and C terminal lobes also move closer together, pivoting about the hinge region, and the T (or activation) loop moves to expose the catalytic cleft. Phosphorylation of Thr 160 further organises the substrate binding site by causing additional changes to the T-loop [59]. The glycine rich loop is very flexible and shows structural heterogeneity in CDK2 structures as well as in protein kinases in general. The CMGC insert (also known as the CDK insert and the MAPK insert) is a flexible region in the C-terminal lobe. See Figure 1 for the locations of the substructures mentioned above.

### Analysis of CDK2 crystal structures

A Polyphony script was used to download the structures of the 95% identity sequence cluster containing PDB code 1HCK, chain A from the RCSB website [61]. At the time of analysis this cluster contained 216 structures and 290 chains. The vast majority were solved with the P2_1_2_1_2_1_ space group and over half had unit cell dimensions of around 53, 69, 72 Å. However, there were examples of 7 other space groups. Starting models with PDB codes were cited for 83 of the structures. Of these, 22 started directly or indirectly from 1HCK or 1HCL [62], 15 from 1FIN [58], 12 from 1QMZ [63] and 12 from 1B39 [64]. There were no other significant groupings of structures reported to start from the same model. Within grouping (‘A’ chains only) average RMSDs to their respective starting model range from 0.2 to 0.5 Å (median 0.2 to 0.6 Å). Overall (again ‘A’ chains only) average RMSDs to these starting structures range from 1.3 to 1.6 Å (median 1.0 to 2.3 Å).

#### The Ramachandran plot redrawn

**Figure 2:**
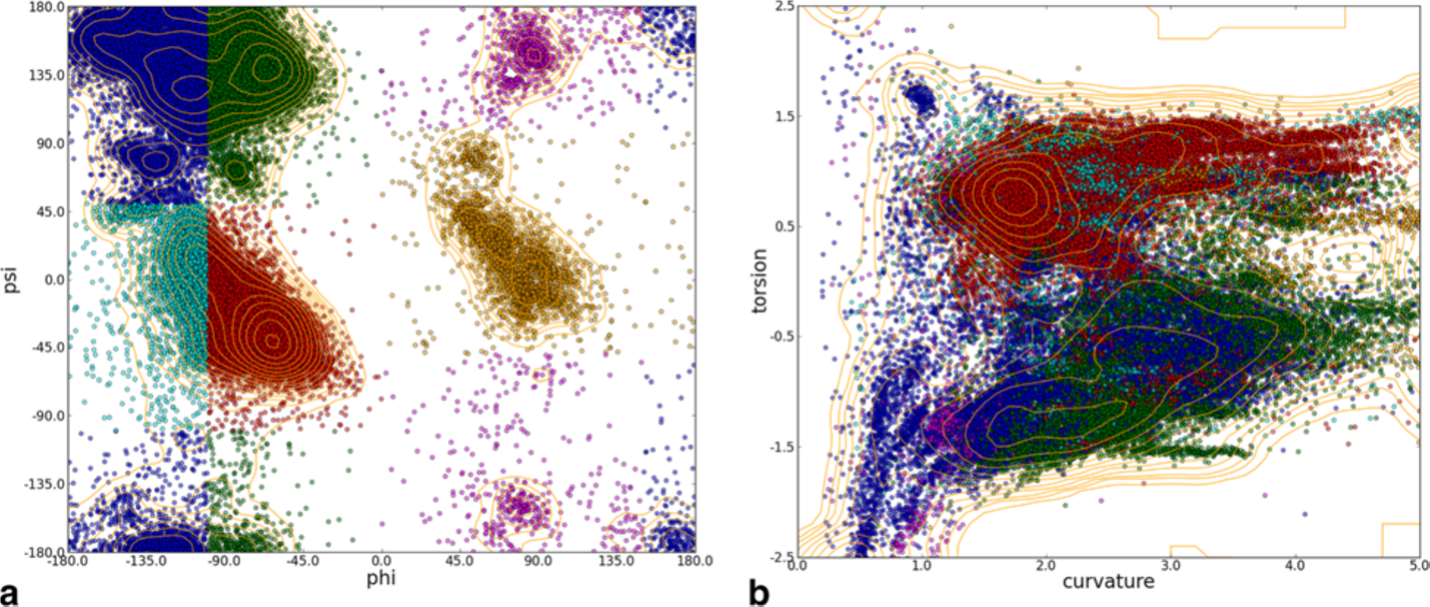
**The Ramachandran plot compared to the plot of residue curvature against torsion. (a)** Ramachandran plot for all residues in all chains. Colours delineate the regions specified in [65]**(b)** Plot of curvature against torsion for each residue in all chains. Colours denote the region the Ramachandran plot shown in **(a)**. Contour levels are at Fibonacci numbers due to the large number of overlapping points.

A Ramachandran plot for all 290 chains combined (Figure 2a) was plotted and separate secondary structure regions colour coded. Figure 2b shows the equivalent plot of curvature against torsion with colours derived from the Ramachandran plot. It can be seen that in this latter plot the residues with the same secondary structure are roughly in the same area of the graph, although these areas are less distinct.

#### Local conformational variability

**Figure 3:**
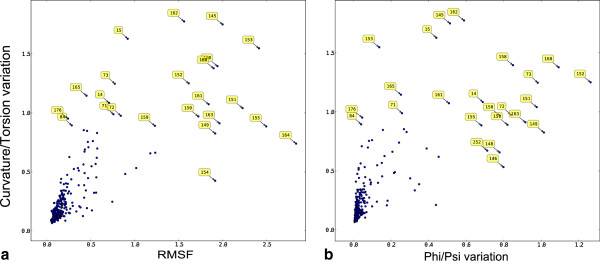
**Cα-trace κ/τ variation compared to two other metrics of backbone variation (a) Root mean squared fluctuation (RMSF) in Cα position after least squares fitting a sliding window of 5 residues to a reference structure and (b) variation in φ/ψ torsion angle (see equation** 1**).** Labels show alignment position numbers indicating that the largest differences lie largely in the T-loop (145–164).

Figure 3a shows how conformational variability for each CDK2 residue calculated from spline κ/τ compares to RMSF. The differences between the two measures increase as variability increases. RMSF is calculated after fitting a sliding window of 5 contiguous residues to equivalents in a reference structure. This fit becomes arbitrary at high degrees of conformational divergence.

Figure 3b shows the conformational variability for each residue calculated from φ/ψ torsion angles and spline κ/τ. Variation is calculated in different ways for the two descriptors (see Methods section) but it is perhaps surprising that they show no correlation. This difference is probably due to the fact that φ/ψ torsion is a more local measure of conformation than κ/τ, which is calculated for a spline whose shape is dependent on the conformation of neighbouring residues. Changes in φ/ψ torsion angles could be neutralised by compensatory changes in the conformation of neighbouring residues.

#### Subgroup comparisons

**Figure 4:**
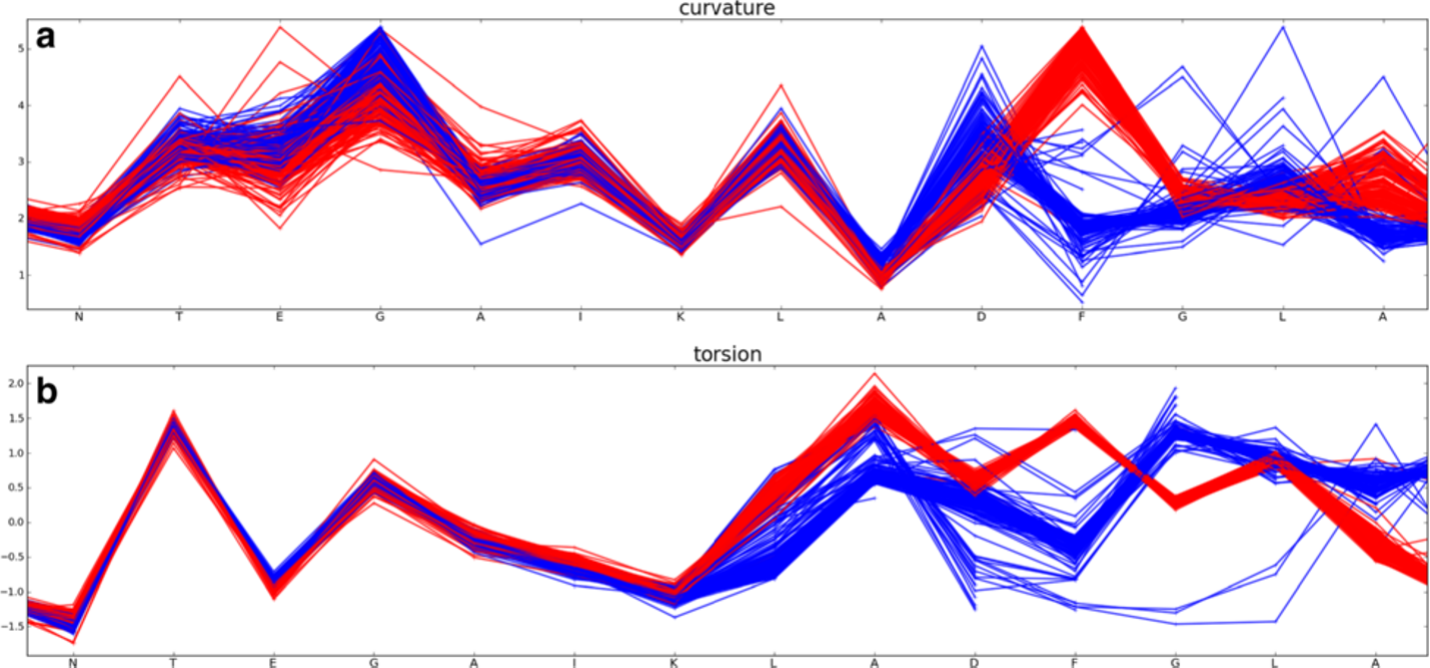
**Plot of curvature and torsion of CDK2 chains as a function of sequence for residues N136 to A149 in the region of the DFG loop.** Monomeric chains are coloured blue and protein-bound chains, red. **(a)** curvature and **(b)** torsion as a function of sequence.

**Figure 5:**
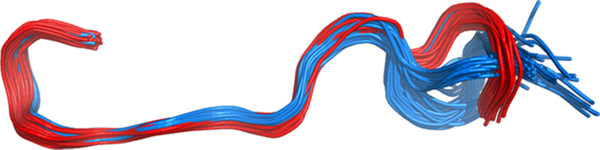
**PyMol [**[45]**] Cα trace for residues N136 to A149 in the region of the DFG loop.** Monomeric chains are coloured blue and protein-bound chains, red.

In order to divide proteins into monomeric and (usually) cyclin bound forms, the chains were clustered using the Tanimoto similarity of the per residue protein-protein interaction fingerprint [66] of contacts extracted from the PICCOLO database [43]. Figures 4a and b below show curvature and torsion values respectively for residues in the region around the conserved DFG motif which lies at the start of the T-loop. The plots are colour coded according to the presence (red) or absence (blue) of a biologically relevant protein-protein interaction. They clearly show the difference in conformation in this region that occurs upon protein binding as shown in 3D in Figure 5.

Note that κ/τ values are not calculated for residues within 2 residues of the termini and the frayed ends adjacent to disordered residues (see Methods).

#### Conformational variability and average temperature factor

**Figure 6:**
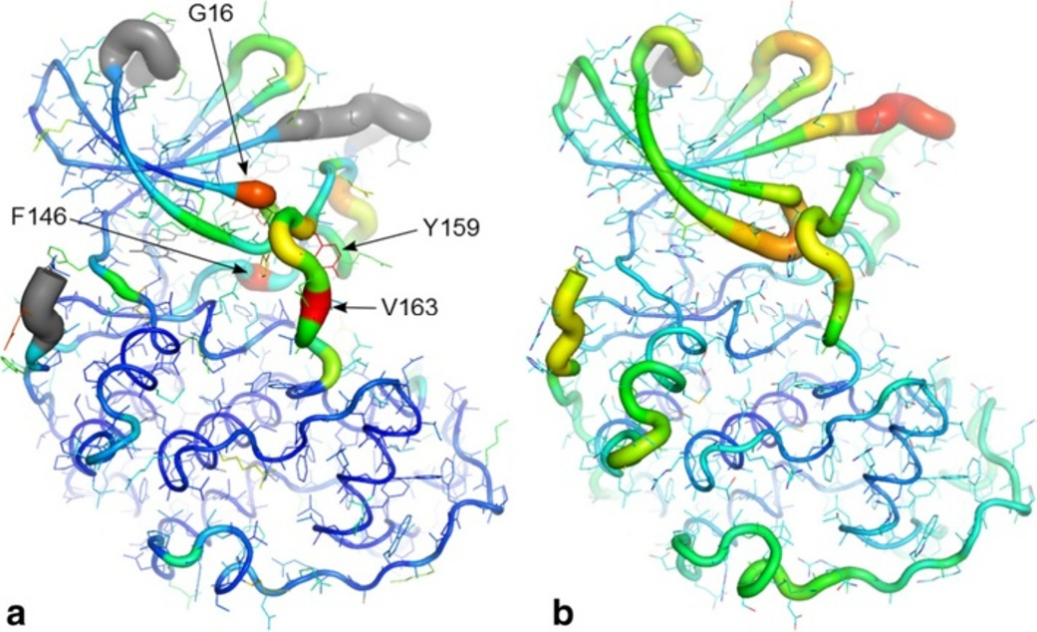
**Conformational variability vs. average temperature factor.** PyMol putty cartoon of 3PXZ [68] chain A showing summaries over 290 CDK2 chains of **(a)** relative variability in conformation of backbone κ/τ (tubes) and sidechains (sticks): a grey colour indicates disordered residues in greater than 50% of structures, and **(b)** average Cα temperature factor (tubes). A red colour and thick tube indicates the highest variability and highest average temperature factor. The labels highlight particularly conformationally variable residues.

Of course loading all 290 chains into a molecular graphics program, aligning them, colouring them etc. is slow, memory intensive, and the results can be messy. Instead, summary views can be generated with Polyphony, where properties of the whole ensemble are projected onto one or more representative structures in PyMol. Figure 6 shows two such summary views. In Figure 6a the per residue relative variability in backbone and side-chain conformation are shown. This can be compared to the relative average normalised Cα temperature factor in Figure 6b. Temperature factor normalisation is done using the per protein mean and standard deviation, calculated after the removal of outlier residues, following the procedure of Smith *et al*. [67]. Relative backbone conformational variability and average temperature factor are grossly similar in distribution along the sequence. However the former is a more local property showing sharper variations. Residues that stand out as having particularly variable backbone conformations are Gly 16, the last glycine in the glycine-rich loop, Phe 146 of the DFG motif and Val 163 near the end of the T-loop. The side-chains of tyrosines 15 and 159 also stand out as conformationally variable. Hinge residues, in which a small conformational change leads to a large shift in a subdomain, are not easily spotted in this analysis.

### The influence of crystal contacts

**Figure 7:**
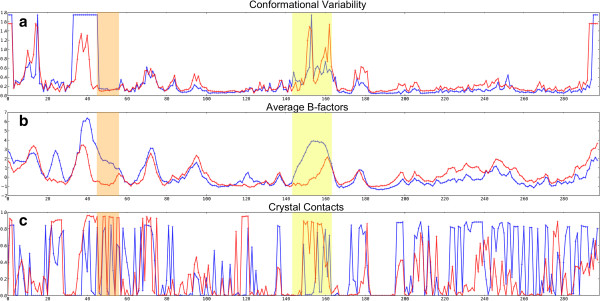
**Comparison of conformational variability, average temperature factor and presence of crystal contacts in CDK2.** Alignment position is labelled on the X axis. Monomeric chains are coloured blue and protein bound chains, red. **(a)** conformational variability using curvature and torsion **(b)** average normalised temperature factor **(c)** crystal contacts as calculated using CCP4 [41] NCONT. The value plotted is the average proportion of structures with at least one contact within 5 Å. The location of the PSTAIRE helix is highlighted in orange and the T-loop is highlighted in yellow.

Viewed in a 2D plot (Figure 7), it is clear that, in the C-terminal half of the protein, after the T-loop, the conformational variability and average temperature factors are higher in the protein bound structures of CDK2. As expected, parts of the T-loop and the loop immediately preceding the PSTAIRE helix are more ordered in the bound form. Enticingly, this presents a picture of a loss of entropy at the protein-protein interface in the T-loop upon binding, which is compensated for by a gain in entropy elsewhere. However, unnatural crystal contacts are also protein-protein interactions and must be taken into consideration. Protein-protein interactions can result in lower temperature factors at the interface [69]. For instance, residues 20–28 have lower average temperature factors in the bound form but probably as a result of conserved crystal contact with another cyclin molecule in the asymmetric unit. Barrett and Nobel [48], using molecular dynamics simulations in solution, found that cyclin bound CDK2 becomes more rigid on phosphorylation, except for the CMGC insert region which becomes more mobile.

#### Hinges and loop flips

**Figure 8:**
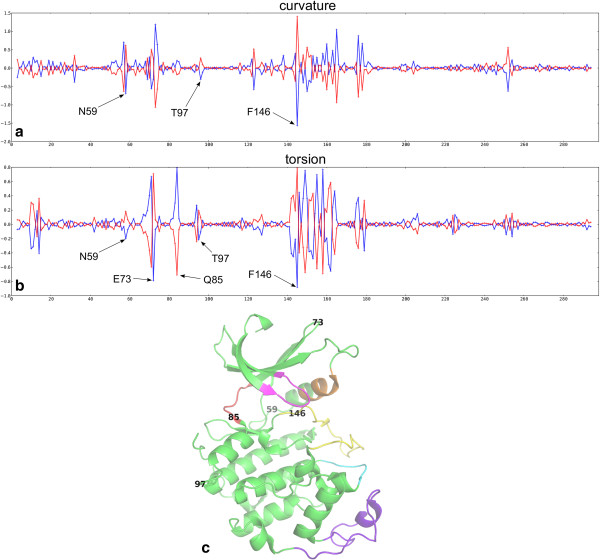
**Plot of alignment position (X-axis) against the group variance** ***s***_***i***_ **(see Equation** **1** **, Y-axis) for monomeric chains (** ***i*** **= 1, blue) and protein bound (** ***i*** **= 2, red) CDK2 (a) curvature** ***s***_***i***_ **(κ) and (b) torsion** ***s***_***i***_ **(τ) (c) positions of the highlighted residues in the structure of CDK2.**

Once groups or clusters of structures are defined, they can be compared in terms of conformation and also interactions. Shown in Figure 8 are the variances (see Methods) in curvature and torsion between bound and unbound CDK2 chains. Interestingly, variances in the curvature and/or torsion at individual residues are sharply defined and not smoothed out due to spline fitting.

It is clear from Figure 8, and from looking at a 3D representation of the data using the Polyphony PyMol API, that hinges are highlighted by this analysis. In particular there is a big change between monomeric and protein bound structure in τ at residue Gln 85 of the hinge region, the κ and τ at Phe 146 of the DFG motif, and a definite signal at Asn 59 at the end of the PSTAIRE helix. In addition many residues light up in the T-loop and the cyclin binding region. This ability to highlight what might be called micro-hinges, which include those between secondary structure elements, as well as between domains, is an advantage of this approach.

Once noise, or random thermal motion, is reduced in this way it becomes apparent that some changes in backbone conformation are compensated for by opposite changes at a neighbouring residue. These paired peaks are the signals produced by loop regions that occupy different but conserved conformations in the groups of proteins (here monomeric and protein bound). Examples that can be seen in Figure 8 are the loops centred at Glu 73 and Thr 97. The Cα trace emerges from the conserved core and bends one way but must then bend back again to return to the conserved core. This sort of conformational change is distinct from a hinge because it only affects local structure. It is worth noting here that, because curvature is always positive (see Methods), a curve to the left (let’s say a positive curvature), which is then compensated by a curve to the right (negative curvature), is not apparent from these plots. Curvature and torsion must be taken together to observe such differences.

#### Principal components analysis

**Figure 9:**
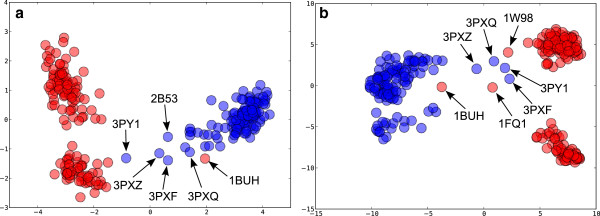
**PCA scores plot for CDK2.** Monomeric chains are coloured blue and protein bound chains red. **(a)** backbone conformation using curvature and torsion. The variance explained by PC1 is 45% and 51% by PC1 and PC2 combined. **(b)** side-chain conformation using the position of a terminal atom in Cartesian coordinates after transformation of the residue to a reference origin (see Methods). PC1 explains 24% and PC1 plus PC2 explains 35% of the variance. Labels show the PDB codes of outliers.

**Figure 10:**
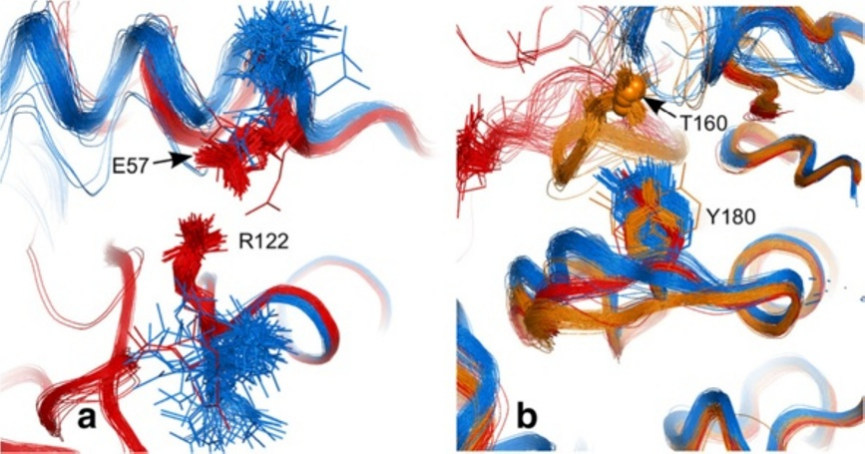
**PCA discriminatory side-chain conformations. (a)** salt bridge formation between Arg 122 and Glu 57 in cyclin bound structures (red). **(b)** Tyr 180 in monomeric (blue), protein-bound but unphosphorylated (red), protein-bound and phosphorylated at Thr 160 (orange, with phosphorus shown as small spheres). The rest of the T-loop is cut away for clarity.

In order to facilitate a principal components analysis (PCA), a full matrix of data was generated (see Methods section). The PCA used the non-linear iterative partial least squares (NIPALS) algorithm implemented in PyChem [70]. The resulting scores plots for backbone and side-chain conformation are shown in Figures 9a and b respectively. It can be seen that the first principal component (PC1) divides monomeric and protein-bound structures. In the backbone conformation PCA (Figure 9a, the loadings plot (not shown) indicates a high contribution from the 72–74 TEN loop tip but also Cys 177, Gln 85 of the hinge region and Asn 59 at the end of the PSTAIRE helix. In the side-chain analysis (Figure 9b), the conformation Arg 122 is the major discriminating factor. It forms a salt bridge with Gln 57 on cyclin binding, seemingly helping to lock the PSTAIRE helix in place (see Figure 10a). The charged residues in this salt bridge are not conserved amongst CDK isoforms.

A structure that stands out as a red dot amongst the blues in Figure 9a, i.e. protein bound structure in amongst the monomers is 1BUH [71]. This structure is a complex of CDK2, not with cyclin, but with cell cycle-regulatory protein CksHs1, which binds at a different site on the C-terminal lobe of CDK2. The chain B of 1FQ1 [72] also stands out in this way in the side-chain conformation PCA plot (Figure 9b). This is the structure of kinase-associated phosphatase (KAP) in complex with phospho-CDK2 (p-CDK2). KAP binds to a different region on the surface of CDK2 than cyclin, but one that overlaps with that of CksHs1. Another structure that stands out is 1W98 [73], which is a structure of p-CDK2 in complex with a truncated cyclin E1. Structures 3PXZ, 3PXQ, 3PY1, and 3PXF [68] lie in the centre of both the backbone and side-chain PCA plots. These are monomeric structures that are located closer to the region occupied by the protein-bound structures. Interestingly, they come from a series of structures in complex with allosteric inhibitors that cause conformational changes to the PSTAIRE helix.

PC2 in Figure 9a and b both subdivide the protein-bound CDK2 structures into two groups. The 179–181 YYS motif, which forms part of the αEF-αF loop (see Figure 1), contributes most to backbone conformation PC2 in the Figure 9a. This loop changes shape on phosphorylation of Thr 160 and is known to be coupled to the activation loops in a variety of kinases [74]. This loop lies underneath the T-loop, which is excluded from the PCA because so many structures (>10%, the default cut-off) are disordered in this region. The side-chain conformation of Tyr 180, in the αEF-αF loop, more or less single-handedly separates structures on the PC2 axis in Figure 9b. Figure 10b shows its different conformations in monomeric, cyclin bound and cyclin bound plus phosphorylated CDK2.

The first two principal components explain a relatively low amount of the variance, especially for side-chain conformations (see Figure 9 footnote). Unlike the usual Cartesian coordinate PCA, we are measuring local changes that act in concert rather than big blocks of secondary structure that move together. Many local conformational states are conserved or change in an uncorrelated manner and therefore do not strongly influence the first principal components.

#### Correlated conformational changes

**Figure 11:**
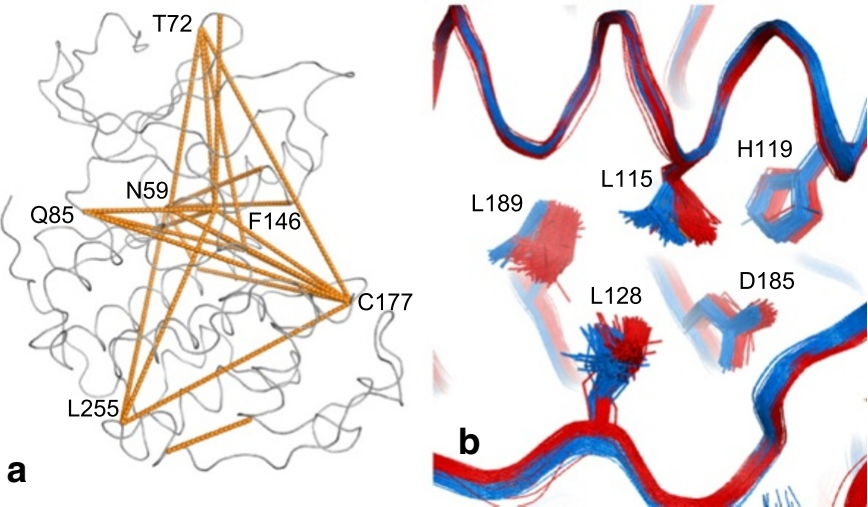
**Correlated conformational change.** The 25 most highly correlated pairs of residues separated in sequence by at least 30 amino acids. **(a)** backbone. The correlation coefficients range from 0.84 between Asn 59 at the end of the PSTAIRE helix and Phe 146 of the DFG motif, to 0.65 **(b)** side-chains. Leu 115 and Leu 189 in the core of CDK2 switch rotamers on protein binding.

In addition to PCA, there is the capability within Polyphony of identifying correlated residue conformational differences between individual pairs of residues. This can be done for backbone or side-chain conformations. The κ/τ and x, y, z correlation coefficients, respectively, are averaged for each residue pair. Lines are drawn between the most highly correlated residues in an analogous way to Young *et al*. [75] and the covariance web produced by Dynamite [23]. These published methods use Cartesian coordinates after structure superposition, whilst here the correlations are calculated for local conformations. Only correlations between residues with a separation of 30 residues in the protein sequence are shown in Figure 11 but this cut-off is under user control in Polyphony.

Most of the CDK2 main chain correlations found in this way are consistent with the results of the PCA and known mechanisms of action but also highlight previously unpublished interrelationships between residues. For instance, Phe 146 of the DFG motif is correlated with Asn 59, identified above as a hinge residue at the end of the PSTAIRE region (correlation coefficient *r* = 0.84). Cys 177 in the αEF-αF loop and Gln 85, which is identified above as the pivotal residue in the hinge region, are also linked to these two residues via a network of correlations. Surprisingly Leu 255 is also paired with these residues, albeit with a lower correlation coefficient (*r* < 0.69) but this probably due to crystal contacts with this residue which are much more common in monomeric compared to protein-bound structures (*cf.* Figure 7c).

Similarly the side-chain conformations of His 268 in the C-lobe are correlated with Asp 68 in the N-lobe (*r* = 0.78). This is almost certainly an experimental artefact, due to crystal contacts (see above). More interestingly, there seems to be a repacking of part of the hydrophobic core on cyclin binding. The neighbouring residues Leu 115 and Leu 189 change conformation in a correlated way (*r* = 0.68) (see Figure 11b).

#### Pocket analysis

**Figure 12:**
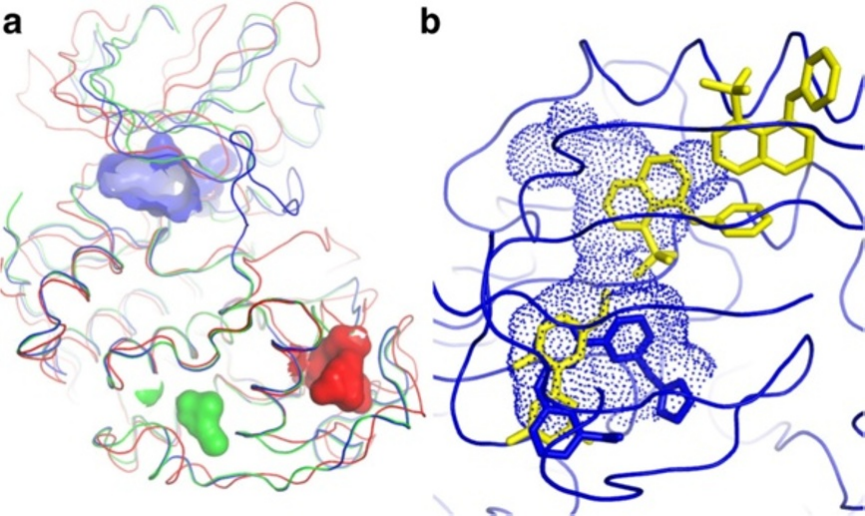
**Location of Fpocket-predicted druggable binding sites selected by the Polyphony distinct pocket selection procedure (n = 3). (a)** The blue pocket was ranked highest and is the ATP binding site in 2C5Y [76] chain A. The second pocket is coloured red and is from 1OI9 [79] chain C. The third in green is from 2R3L [80] chain A. **(b)** the blue pocket from (a) viewed from above, overlaid with a recently discovered allosteric ligand from 3PXZ [68]. The ligand from 2C5Y is shown in blue sticks.

The pocket detection program Fpocket [32] was chosen for integration with Polyphony because it is an open source program and it provides a druggability score for each pocket. However, it is possible to integrate other programs because of Polyphony’s plug-in architecture. As employed by Provar [33], the assignment of pocket attributes to residues facilitates the comparison of structures of the same or homologous proteins, given a sequence alignment. Like Provar, the fraction of structures in which a particular residue (or alignment position) is found to be part of a pocket are reported, here expressed as a percentage. Again like Provar, these percentages are mapped onto the coordinates of a representative structure as colour-coded surfaces. In Polyphony there is a novel methodology which shows the user distinct (but sometimes overlapping) predicted druggable pockets in the structure in which they occur (see Methods section). In this way cryptic pockets can easily be identified and visualised. Figure 12 shows the results of this procedure applied to the CDK2 structures. The highest ranked pocket (cyan in Figure 12) was an ATP binding pocket which is connected to a water filled cavity behind the PSTAIRE helix in 2C5Y [76]. This cavity would not exist when the PSTAIRE helix moves in on binding to cyclin. Thus this cavity is a potential binding site for allosteric inhibitors of cyclin binding. In fact, fragments that bind in this cavity were recently discovered in later published structures [68]. The pocket selected second (magenta in Figure 12), which lies under the CMGC insert also shows potential as a binding site for allosteric modulators. The region (also known as L14 in CDK2) is known to be flexible from simulations and analysis of crystallographic temperature factors [48]. It forms part of the binding site for Cell Cycle–Regulatory Protein CksHs1 [71] and kinase associated phosphatase (KAP) [72]. There are no small molecule ligands bound to this pocket in the CDK2 crystal structures but there are in p38α [77] and JNK-1 [78]. The third pocket identified (yellow in Figure 12) is close by in the C-lobe.

#### Intermolecular interactions

Atomic scale interactions are extracted from three different sources 1/ The PICCOLO database [43] of predicted biologically relevant protein-protein interactions 2/ The CREDO database [42] of protein - small molecule interactions and 3/ Crystal contacts calculated using the CCP4 [41] program NCONT. This program is not open source but is available without cost to academic and non-profit institutions. The two data sources are MySQL databases created in-house. PICCOLO is currently being updated monthly and can be downloaded and installed locally for free. The original CREDO was frozen in April 2010 and replacement by a new PostgreSQL database [81]. Residue-based structure interaction fingerprints (SIFts) [66] where extracted from PICCOLO and CREDO and analysed in various ways including clustering (*cf.* clustering of CDK2 structures into monomeric and protein-bound above) using Tanimoto similarity.

Two papers have explored the differences in inhibitor binding in CDK2 ATP binding site between active and inactive forms, by comparing two [82] and twelve [76] structures. A comparison of CREDO derived SIFts for the 250 protein-bound and monomeric CDK2 chains with ligands bound, revealed that the set of residues interacting with small molecule ligands were almost identical, except for Glu 51. This residue is part of the PSTAIRE helix and the conserved catalytic triad and swings into the ATP binding site on activation [58]. None of the 134 ligands bound to monomeric CDK2 contact this residue (distance cutoff 4.5 Å), whereas 55 of the 116 (~50%) bound to protein complexes do.

#### Sequence alignment view

**Figure 13:**
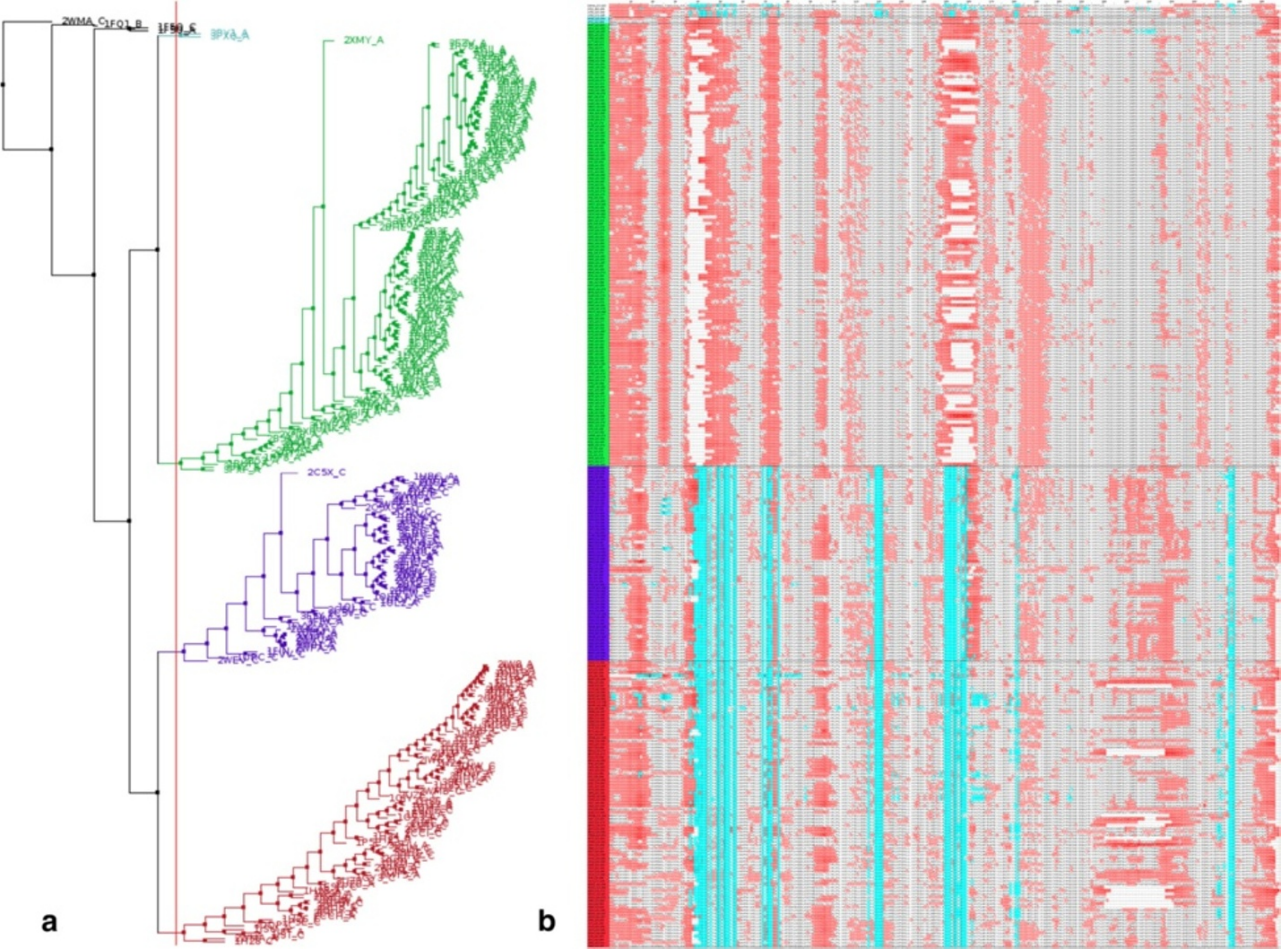
**A sequence alignment sorted and annotated by structural and conformational features.** **(a)** dendrogram and **(b)** sequence alignment view of CDK2 structures shown in Jalview [46]. The alignment is ordered by the tree, generated by hierarchical clustering using backbone curvature-and-torsion Pearson similarity. The red vertical line in the dendrogram cuts the chains into 3 large clusters, which correspond to monomeric (green), cyclin bound (purple) and cyclin bound and phosphorylated (red). Amino acids in the alignment are colour-coded by B-factor (red) and protein-protein contacts from PICCOLO [43] (cyan). White gaps indicate disordered residues.

Sequence alignment viewers are, of course, a very useful way of visualising similarities and differences in the amino acid sequences of related proteins. In many cases the simple single letter amino acid codes are augmented with colours to indicate a particular property of each residue such as hydrophobicity or known features such as the occurrence of a post translation modifications. This idea has been extended to include descriptions of the residue conformation or environment with the folded protein [83]. Here we are concerned, in the main, with multiple structures of the same protein. Thus, a plain sequence alignment is not very informative. However once annotations, such as colour coded B-factors and protein-protein contact counts, are added, the sequence alignment view becomes a convenient way to visualise the similarities and in conformation and interactions across a large number of structures. Jalview [46] was chosen for this purpose because users can import colour-coded residue features from a text file. It also allows the import of Newick format trees, which can then be used to sort sequences. Polyphony can create Jalview feature and Newick tree files for any of the residue-based properties. Figure 13 shows what this looks like.

### Application to the analysis of homologous proteins

Even when only a handful of structures of a given protein are available they can be usefully compared with those of homologous proteins. Differences between the structures of homologous proteins include architectural as well as conformational changes. To separate out these two sources of variation, in Polyphony, conformational variance is calculated separately by protein. Then the locations of intra-protein variance are compared across homologous proteins. Below this type of analysis is illustrated on members of the CMGC family of protein kinases [84]. A structural alignment was taken from the HOMSTRAD database [85].

**Figure 14:**
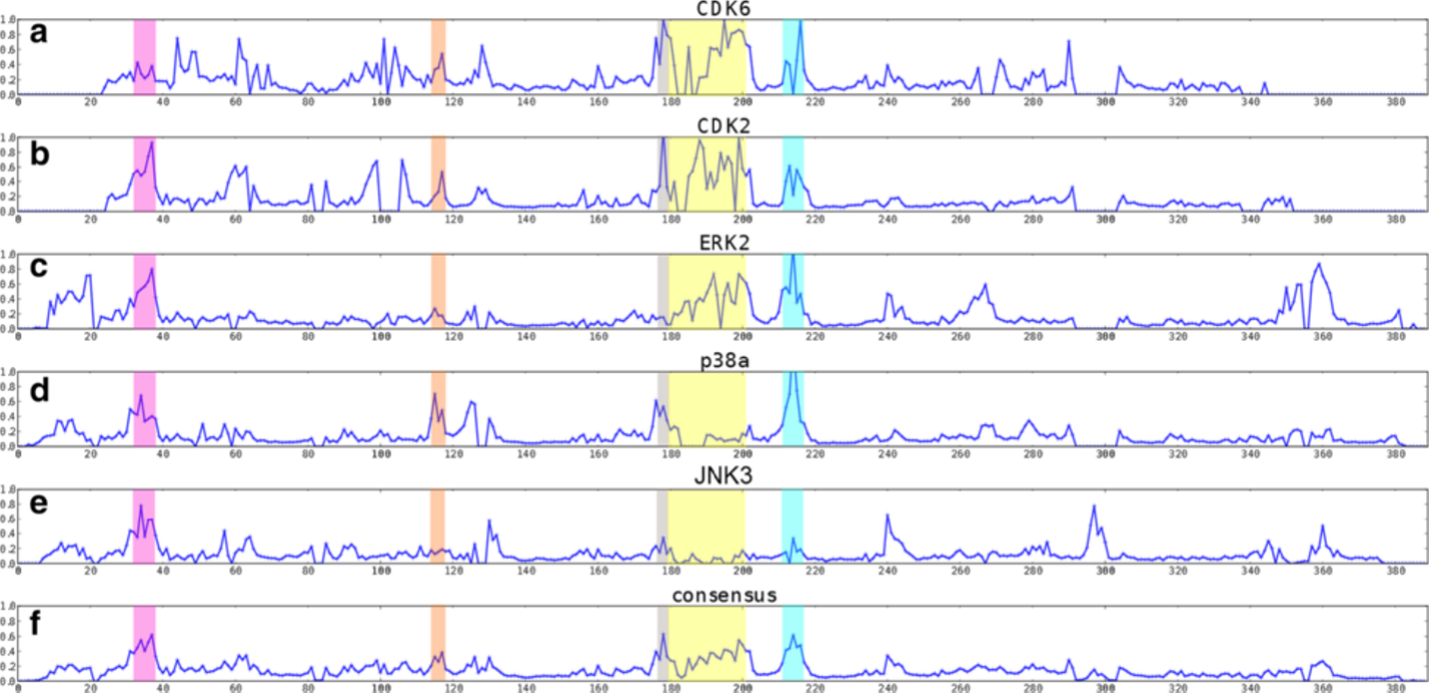
**Plot of the group variance** ***S*** **(see Equation** 5)**for CGMC protein kinases.** Groups were clusters of very closely related structures (1% Pearson distance cutoff). **(a)** CDK6, n = 12 in 9 groups **(b)** CDK2, 290 in 93 **(c)** ERK2, 27 in 18 **(d)** p38α, 186 in 75 **(e)** JNK3, 32 in 22 **(f)** consensus (average *S*). The colours indicate the glycine rich loop (pink), the hinge region (orange), the DFG motif (grey), the T-loop (yellow) and αEF-αF loop (cyan).

Figure 14 shows the aligned variance plots for CDK2, ERK2, p38α and JNK3. The regions of known conformational change are highlighted. The glycine rich loop has high values in all proteins. A peak is visible in the hinge region, particularly in CDK2 and p38α. The DFG motif has a peak in all proteins except ERK2. The activation loop has a high peak in the CDKs and ERK2. There also peaks in the consensus plot in lesser-known regions, particularly in the αEF-αF loop region.

Tyr 180 (in the αEF-αF loop region) in CDK2 interacts with the phosphorylated Thr 160 and has distinct conformations in monomeric, cyclin-bound and cyclin-bound plus phosphorylated forms (see side-chain PCA analysis above and Figure 10). In p38α the αEF-αF peak occurs at Met 198. In contrast to CDK2 Tyr 180, this residue is not in close proximity to the phosphorylated residues Thr 180 and Tyr 182. However, its backbone conformation is strongly correlated with residues 180–184 (*r* = 0.63-0.75). It takes two main conformations, one where the side-chain is surface exposed and one where it is buried under the CMGC insert. The insert itself shifts as result and is 2 or 3 Å closer in to the rest of the protein in the latter conformation. As mentioned in the Pocket analysis section above, this region has recently become the focus of inhibitor discovery and design in JNK-1 and p38α [78]. In ERK2 it was recently discovered that the CGMC insert, which is a helix-turn-helix motif, is involved with nuclear localisation [86] and even DNA binding [87]. Our analysis reveals what appears to be an evolutionary conserved link between phosphorylation and the conformation and dynamics of this functionally important part of these proteins.

### Use on computed conformations

**Figure 15:**
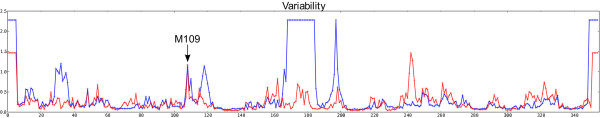
**Backbone conformational variability for X-ray structures (blue) and an MD simulation of p38 (red).**

One of the aims of this project was to create new ways to compare experimental structural ensembles with those generated by computational methods. As shown in Figure 7, variability of backbone curvature and torsion provides a more local measure of conformational change than average B-factor. Figure 15 shows variability over 186 p38α X-ray chains and a MD simulation downloaded from the MoDEL database [88]. The simulation was of the structure 1A9U [89] for 12 ns. For this analysis 180 snapshots separated by 50 ps, covering the last 9 ns of the simulation, were extracted as a multimodel pdb format file. Many of the conformational changes seen amongst the crystal structures are likely to occur on a much longer timescale than that covered by the simulation. However there is some qualitative similarity in the variability observed in these structures and those generated by the simulation. For instance, the change in conformation at the key hinge residue Met 109 is reproduced, indicating that this is a due to a high frequency motion. There is also clearly some dynamics in the αEF-αF loop centred on Met 198, which is highlighted in the previous section as being a region of significant conformation heterogeneity. This residue remains buried under the CGMC insert during this short simulation but the αEF-αF loop appears to stretch out as the CGMC moves away from the C-terminal lobe as a whole. There are also discrepancies between the crystal structure conformational difference and the simulated dynamics. For instance, in the region 240–245 there is partial unravelling of the first helix of the CGMC insert in the simulation, whereas it is well conserved between crystal structures. This could be due to the influence of crystal contacts or other reasons. Work is under way to further assess the use of Polyphony for the analysis of MD trajectories.

## Conclusions

The suite of new methods for the analysis of ensembles of protein structures is selected on the premise that there are advantages to comparing structures without superimposing them. The approach taken is analogous to sequence analysis techniques and starts from a sequence alignment. The alignment itself is trivial because the sequences involved are almost identical. The amino acid types in the columns in the alignment do not differ (point mutations excepted) but the conformations of the residues do. Backbone conformational changes can be those that have very little effect on the shape of the protein, such as those near the termini or at the end of flexible loops. Others are hinging motions that change the relationship between domains. One difference between these two extremes of variation is that the former tend to be randomly distributed and the latter tend to be consistent across multiple structures. Another difference is that the latter tend to coincide with environmental changes, such as a binding event. When a binding event is a protein-protein interaction with a large interface, correlated conformational changes tend to occur at the interface and allosterically. All of these effects can be detected by statistical analysis of all the local conformational differences within an ensemble without the need to superimpose the structures and calculate Euclidean distances in Cartesian space. Once discovered in this way, the consequences of these significant conformational changes can be observed by visual comparison of the structures and the use of existing analytical techniques. The obvious drawback with this approach is the requirement for a large sample size. One must also be wary of systematic experimental artefacts such a conserved non-biological crystal contacts. These issues can be addressed by complementing X-ray structures with NMR derived and computationally generated ensembles. However, there is great benefit not only in solving the crystal structures of new proteins but also in solving the structure of same protein multiple times, especially when co-crystallised with new binding partners.

Tools and methods for superposition-independent statistical analysis of protein structure ensembles were developed. The general approach, and the individual methodologies within it, were validated by the rediscovery of the published findings of the many authors who have compared crystal structures of CDK2 since Jeffrey *et al.* in 1995 [58]. The major conformational changes that occur on cyclin binding, such as the movement of the PSTAIRE helix and the opening and closing of the gap between the N and C-terminal lobes, were detected via symptomatic changes in hinge residues. In addition, more subtle changes were also detected and found to be conserved in other CMGC kinases. These include correlated changes in the αEF-αF loop linking phosphorylation sites on the activation loop to the CMGC insert. This information provides a further clue to the role of this region whose importance is only recently beginning to be revealed and targeted with small molecule ligands in JNK3 and p38α. Thus far no structures of CDK2 with small molecules bound in this region have been published. The pocket analysis above reveals a pocket can exist there and it is predicted to be druggable, illustrating the utility of ensemble-based drug discovery.

## Methods

### Implementation

Polyphony is implemented as Python modules and example scripts. It uses Biopython [90] for PDB and sequence alignment file parsing. The core data structure is the NumPy masked array [91] which is ideal for handling gaps in alignments due to, for example, unstructured residues. SciPy [92] modules are also used extensively. Graph visualisation is achieved with matplotlib [44]. Sequence alignment feature viewing is done with Jalview [46] via Polyphony-generated feature files and Newick format tree files. There is also an extensive application-programming interface (API) for PyMol [45] for 3D visualisation using the built-in XML-RPC server which can be used in combination with IPython [93]. The philosophy employed is to avoid re-inventing tools if there is already something useful that is freely available. To this end, parsing routines are sometimes used to facilitate the translation of output from 3rd party programs into Polyphony objects. These programs include Fpocket [32] for pocket finding, ETE [47] for interactive tree diagrams, CCP4 [41] NCONT for crystal contact counts, and the PyChem [70] mva module for multivariate statistics. With the exception of PyChem, these programs must be downloaded and installed separately by the user if they wish to use them. For intermolecular interactions, the databases PICCOLO [43] and CREDO [42] developed within the Blundell group are queried using via SQLAlchemy [94] or the credoscript Python API. Documentation was written with the help of Sphinx [95]. There is a Bitbucket [96] repository and code versions are managed using Mercurial [97].

### Code architecture

Polyphony has an object-oriented structure with classes for manipulating the structural alignment, for calculating and for comparing the properties of the protein structures. These latter two classes inherit the ability to store and re-use the calculated data automatically from a data management class. This feature facilitates interactive analysis, for instance using the PyMol API. Each property calculation method is contained within its own subclass. A configuration file controls the selection of these subclasses. This plug-in type architecture allows methods that depend upon 3rd party software to be deselected if the user doesn’t wish to install them and new custom built methods to be introduced seamlessly.

### Description of backbone conformation

**Figure 16:**
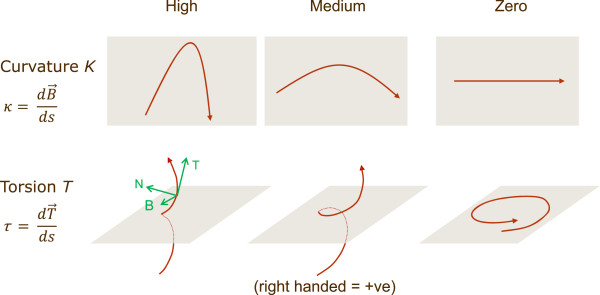
**Curvature and torsion.** Curvature measures the deviation from a straight line and torsion measures the deviation from a plane curve [98]. T, N and B are the tangent, normal and binomial (the cross product of T and N) of the Frenet-Serret frame. s is the arc length along the spline.

In a similar way to CHORAL [37], the curvature (κ) and torsion (τ) (see Figure 16) at each Cα atom are calculated for a B-spline fitted through the Cα atoms of each protein chain. These values are standardised over all residues in a structural alignment and capped at 3 standard deviations from the mean. The conformation of protein chains is compared using the Pearson distance ((1 - Pearson correlation)/2.0) which is sensitive to outliers, hence the capping.

Curvature is always positive. Although a signed curvature can be defined for a curve in the 3-dimensional Euclidian space [35], by embedding it into a surface, the added data are not relevant as it already contained in the additional information supplied by the torsion [99]. It is a well known result from differential geometry that any curve in space can be completely defined by its curvature and torsion alone. For most cases a signed curvature is only defined for curves on a plane as they naturally present zero torsion and, in this case, the added data could be relevant.

### Side-chain conformation

The conformation of side-chains is modelled very simply as the relative position of a sentinel atom near the terminus of each side-chain. Atom types that can be assigned unambiguously in protein X-ray crystallography are chosen by preference to avoid artifactual differences between structures. In the cases of valine, threonine and histidine, the atom T is chosen (between two ambiguous choices) such that C-Cα-Cβ-T pseudo dihedral angle is positive. The x, y, z of these sentinel atoms are recorded after the fitting the N-Cα-C-Cβ atoms to reference atoms at the origin. Gly and Ala residues are masked.

### Conformational variability

Using the curvature and torsion values described above, backbone conformational variability over a number of structures can be calculated per alignment position. Variability is defined as the average Euclidean distance from the median values for curvature and torsion. Similarly, for side-chain variability, the same equation is applied to the x, y, z coordinates of the transformed sentinel atoms. If the number of structured residues falls below a given percentage then the variability measure is masked for that alignment position. For φ/ψ dihedral angles, the order parameter of Hyberts *et al.* [100] was used to calculate variability according to equation ( [1]).
12

Where *n* is the number or structured residues at an alignment position for which φ/ψ torsion angles have been calculated.

### Identifying significant conformational differences

The analysis below is designed to separate high frequency thermal motions from more significant changes that occur over longer timescales. It is also used to find the conformational changes that accompany an environment change, for instance the binding of a ligand. In the former case, structures are collected together into groups of closely related structures. The assumption is made that changes between very closely related structures, which are relatively small by definition, are less significant. In the latter case, the structures are split into two groups e.g. *apo* and *holo* forms. The equation (3) is used as a measure of the grouped variance.
3

Where *i* is a group, *j* is a member of group *i* and *n*_*i*_ is the number of members of group *i*, *x*_*i,j*_ is a conformational descriptor of residue *j* in group *i*. Missing values of *x*_*i,j*_ are masked.  is the average of all non-missing values in an alignment position.
4

Where *n* is the number of groups, *N* is the total number of non-missing values. *S* is a statistic describing the significance of the backbone conformational change at each alignment position in a single number. Equation 5 shows how curvature and torsion variance are combined.
5

### Identification of distinct druggable pockets within an ensemble of structures

Fpocket [32] is run on each structure in the ensemble and each residue is assigned a Dscore (Fpocket’s druggability score [101]). This creates an n by m matrix of Dscores, where n is the number structures and m the length of the alignment. The algorithm below is used to process this matrix.

1. For each alignment position, find the residue with the highest Dscore. Ignore residues with a Dscore below a certain cutoff (0.9 by default^*^). Because all residues that belong to the same Fpocket pocket have the same Dscore, whole pockets are selected by this procedure.
2. Sort structures by decreasing number of highest D scoring residues. Keep only top t number of structures (t = 5 by default). This, in effect, sorts structures by pocket size.
3. Each residue selected in stage 1, and found in one the structures selected in stage 2, is assigned a number from 0 to t - 1. All other alignment positions are ignored. The result looks like this for the CDK2 example : [- - - - - - - - - 0 4 - - - - - - 0 - - - - - - - - - 4 4 4 0 4 0 - - - - - - - - - - - - - - - - - - 0 - - 0 4-0 0 0 0-0 0 0 0 4 - - - - - - - - - - 0-0 0 0 0 - - - - - - - - - - - - - - - - - - - - - - - - - - - - - - - - - - - - - - - - - - - - - - - 0 0-0 - - - - - - - 0 0 0 0 0-0 - - - - - - - - - - - - - - - - - - - - - - 1 - - 1 1 - - - - - - - - - - - - - - - - - - 2 - - - - - - - 2 2 - - - - - 1 - - 1 1 - - 1-2 2-1 1 - - - 3 1 - - 1 - - - - - 1 - - - 1 1-1 - - 2–2 - - 2 2 - - 3 - - - - 3 3-2 3 3-2 3 1 - - - - - - - - - - - - - - - - - - - - - - - - - - - - -].

^*^ The authors of Fpocket found that a cut-off of 0.7 was best for identifying druggable pockets [101]. Here a higher cut-off produced more meaningful results for CDK2, probably because the high number of structures used meant that the chances of finding pockets with a higher Dscore were increased.

For display purposes a PyMol surface is created for only the original atoms labelled by Fpocket as belonging to the selected pockets in the selected structures. The PyMol surface setting “Cavity and Pockets (culled)” gives the best results.

### Generation of a full data matrix

Some methods are not suitable for incomplete data matrices. Since missing residues are common in protein crystal structures, due to disorder, a method of selecting a complete submatrix was employed. It is a simple, non-optimal solution to this problem and is described below. It’s based upon an initial removal of the most disordered structures, followed by the removal of alignment positions containing the most gaps.

1. The maximum percentage of chains (*mpc*) to ignore is defined (default *mpc* = 10%).
2. Repeat on alignment column *i* in order of decreasing number of gaps
  - if number of chains to be removed exceeds *mpc*, exit loop
  - remove all chains with a gap in column *i*.
3. Remove all columns that contain a gap.

### Availability and requirements

**Polyphony homepage:**http://wrpitt.bitbucket.org/polyphony/

**Operating system:** Linux

**Programming Language:** Python 2.6, 2.7

**License:** GNU GPL

## Abbreviations

NMR: Nuclear magnetic resonance
PDB: Protein Data Bank
API: Application programming interface
PCA: Principal components analysis
NIPALS: Non-linear iterative partial least squares
Κ: Curvature
Τ: Torsion
RMSD: Root mean squared deviation
RMSF: Root mean squared fluctuation.
